# Mediating effect analysis of postprandial triglyceride on Omega-3 and nonalcoholic fatty liver disease in individuals with normal fasting lipid levels

**DOI:** 10.3389/fphys.2026.1742536

**Published:** 2026-01-16

**Authors:** Luxuan Li, Yale Tang, Yilin Hou, Xiaoyu Wang, Dandan Liu, Peipei Tian, Guangyao Song

**Affiliations:** 1 Department of Internal Medicine, Hebei Medical University, Shijiazhuang, Hebei, China; 2 Department of Endocrinology, Hebei General Hospital, Shijiazhuang, Hebei, China; 3 Department of Endocrinology, Baoding First Central Hospital, Baoding, Hebei, China

**Keywords:** mediating effect, NAFLD, Omega-3 levels, oral fat tolerance test, postprandial triglyceride levels

## Abstract

**Background:**

The association between Omega-3 (ω-3)and non-alcoholic fatty liver disease (NAFLD) in individuals with normal fasting lipid levels subjects is unclear. In addition, few studies have explored whether postprandial triglyceride levels (PTG) mediates the association between ω-3 and NAFLD. We aimed to analyze the mediating effect of PTG on ω-3 and NAFLD.

**Methods:**

In March 2024, volunteers were recruited from the Hebei Provincial People’s Hospital. In total, 108 volunteers met the inclusion criteria. The basic information and biochemical parameters, as well as ω-3 and PTG were collected. NAFLD was diagnosed according to abdominal ultrasonography. The clinical characteristics of the participants was analyzed by quartiles of ω-3 (O1-O4 quartiles) and PTG (P1-P4 quartiles), respectively. Pearson correlation analysis was performed to investigate the correlation between ω-3 and PTG. Multivariate logistic regression analysis was applied to analyze the effect of ω-3 and PTG on NAFLD. Bootstrap was conducted to explore whether PTG mediated the association between ω-3 and NAFLD.

**Results:**

Pearson correlation analysis indicated that ω-3 was negatively associated with PTG. Multivariate logistic regression analysis indicated that compared to the low ω-3 group, the risk of NAFLD significantly decreased in high ω-3 group [OR = 0.024 (0.006 ∼ 0.104)]. Mediating effect analysis showed that ω-3 significantly directly influenced NAFLD prevalence [β = −0.077, 95%CI (−0.128, −0.026)], and PTG partly mediated the indirect effect of the ω-3 on NAFLD prevalence [β = −0.084, 95%CI (−0.130, −0.037)], and the mediating effect accounted for 52.17% of the total effects.

**Conclusion:**

In this cross-sectional analysis, both ω-3 and PTG were predictors of NAFLD, and PTG partly statistically mediated the indirect effect of the ω-3 on NAFLD prevalence.

## 1 Introduction

NAFLD is a group of diseases characterized by liver cell damage and fat deposition in the liver tissues, posing a significant threat to human health (Anwar et al., 2023). Currently, no specific drugs are currently available for the treatment of liver lipid deposition.

In recent years, more attention has been paid to omega-3 polyunsaturated fatty acids, due to their multiple roles in promoting health and reducing disease risks. Studies have found that the incidence of NAFLD is related to a reduction in liver ω-3 (Hong et al., 2019). This suggests that an imbalance in the ratio of ω-3 may be involved in NAFLD. However, there are few related studies on the Chinese population.

In recent years, with improvements in living standards, the prevalence of dyslipidemia in China has significantly increased (Gao et al., 2025). Before 2009, the guidelines and consensus recommended using fasting lipids to assess lipid metabolism status; however, because individuals spend most of the day in a postprandial state, fasting measurements provide only a partial assessment. Since 2009, the guidelines and consensus in many countries have recommended a combined screening of postprandial lipids (Downs and O'Malley, 2015; Nordestgaard et al., 2016; Liu and Zhao, 2021; Catapano et al., 2017). Recent studies have shown that PTG was a strong predictor of NAFLD risk (Griffin, 2015).

However, few studies have explored whether PTG mediated the association between ω-3 and NAFLD. Therefore, this study aimed to investigate the association between ω-3 and NAFLD. Simultaneously, the mediating effect of PTG on the association between ω-3 and NAFLD was analyzed to provide new ideas and more clinical evidence for early identification and intervention of NAFLD.

## 2 Materials and methods

### 2.1 Research participants

This study was conducted in strict accordance with the Declaration of Helsinki. It was approved by the Ethics Committee of the Hebei Provincial People’s Hospital (Approval Research Ethics Review 2024-LW-042) and registered in the Chinese Clinical Trial Registry (ChiCTR2100048497). From March to June 2024, eligible volunteers were recruited from the outpatient department of Hebei Provincial People’s Hospital. All volunteers signed an informed consent form and completed a questionnaire (including basic information, personal history, family history, and medication use) as required. The volunteers were aged 18–60 years.

The first group of exclusion criteria included:

History of fainting during blood injection or blood transfusion and inability to undergo multiple venous blood collections.

History of food or drug allergies and intolerance to high-fat or high-protein foods.

History of diabetes, cardiovascular or cerebrovascular diseases, kidney diseases, blood diseases, infectious diseases, mental disorders, and malignant tumors, among other conditions; pregnant or lactating women; history of severe infections, surgeries, or trauma.

Currently taking medications that affect glucose and lipid metabolism (hypoglycemic drugs, lipid-lowering drugs, contraceptives, hormones, β-blockers).

Currently participating in other clinical trials.

The second exclusion criterion:

All the participants underwent fasting blood glucose (FBG), fasting lipids, and HbA1c tests. According to the Chinese Guidelines for the Prevention and Treatment of Type 2 Diabetes released in 2020 (Chinese Diabetes Society, 2021), patients with diabetes (fasting venous blood glucose ≥7.0 mmol/L and/or HbA1c ≥ 6.5%) were excluded.

To avoid the influence of abnormal fasting lipids on postprandial lipid metabolism, according to the Chinese Lipid Management Guidelines published in 2023 (Joint Committee on the ChineseGuidelines for Lipid M, 2023), participants with fasting triglycerides (FTG) ≥ 1.7 mmol/L and/or fasting total cholesterol (FTC) ≥ 5.2 mmol/L were excluded.

### 2.2 Definition of NAFLD

Clinical diagnostic criteria for NAFLD: (1) No history of alcohol intake or weekly alcohol intake <140 g in males and <70 g in females. (2) The liver imaging diagnosis was consistent with diffuse fatty liver after excluding other causes. Ultrasound imaging diagnosis: (1) The liver near-field echogenicity is enhanced diffusely (“bright liver”) and is stronger than that of the kidney; (2) The structure of the intrahepatic duct is unclear; (3) The liver far-field echogenicity decreased gradually. NAFLD was defined by at least two positive ultrasound findings above (Fatty Liver Expert Committee CMDAFatty LiverExpert Committee and Chinese Medical Doctor Asso ciation, 2018).

Abdominal ultrasound examinations are conducted by doctors with a title of associate chief physician or above. There exists a closed-loop quality management system to ensure inter-rater reliability assessments. Ultrasound images were analyzed by experienced readers who were blinded to the participants’ treatment group and all laboratory metabolic data.

### 2.3 Collection of basic information

General information on the participants was collected, including height, weight, waist circumference, hip circumference, and blood pressure. All measurements were recorded uniformly by the same physician using the same equipment. Body mass index (BMI) was calculated as weight (kg)/height^2^ (m^2^).

### 2.4 Blood tests

Biochemical indicators, such as liver and kidney function, TC, TG, HDL-C, LDL-C, and blood glucose levels at 0, and 4 h of the OFTT were all measured using a fully automatic biochemical analyzer produced by the Hitachi Company. The non-HDL-C levels is calculated using the following formulas: non-HDL-C = TC - (HDL-C). Fasting insulin (FINS) was detected using electrochemiluminescence. Insulin resistance was evaluated using the steady-state model, HOMA-IR = FBG (mmol/L) × FINS (μIU/mL)/22.5 (Matthews et al., 1985). The fatty acid content of the participants was quantitatively detected using gas chromatography-tandem mass spectrometry.

The OFTT was optimized in preliminary experiments performed by our research group. A fixed-calorie high-fat mixed meal of 700 kcal was prepared with the ratio of fat: protein: carbohydrate being 60:25:15%. The fat was provided through Ferica TM(saturated fatty acid:medium-chain fatty acid:monounsaturated fatty acid:polyunsaturated fatty acids = 16.7:13.9:24.6:12.5), the protein through Protein Supplement TM whey protein powder, and the carbohydrates through a 50% glucose injection solution. The components were mixed to form a 300 mL aqueous solution. Before the experiment, a 1-week washout period with a normal diet was required. During the experiment, smoking and drinking were prohibited and strenuous exercise was not allowed. After fasting for more than 8 h, on the day of the experiment, and after collecting the fasting blood samples, a 700 kcal fixed-calorie high-fat mixed meal was consumed by the participants. They all finished the meal within 10 min and did not eat or drink anything else for the next 4 h except for water, which they could drink freely. Blood samples at 0 and 4 h after the meal were collected from the elbow vein using a puncture and indwelling needle for detection.

### 2.5 Stratification at Omega-3 levels and postprandial triglyceride levels

Omega-3 was divided into O1∼O4 groups according to quartiles: O1 group ≤86.35 μmol/L, 86.35 μmol/L < O2 group ≤98.62 μmol/L, 98.62 μmol/L < O3 group ≤121.55 μmol/L, O4Group>121.55 μmol/L.

We define O1 group + O2 group as low ω-3 group, O3 group + O4 group as high ω-3 group.

PTG was divided into P1∼P4 groups according to quartiles: P1 group ≤1.195 mmol/L, 1.195 mmol/L < P2 group ≤1.650 mmol/L, 1.650 mmol/L < P3 group ≤2.325 mmol/L, P4group>2.325 mmol/L.

### 2.6 Statistical analysis

SPSS 26.0 software and incorporate PROCESS v4.2 macros was used for data analysis. Continuous variables with normal distribution were expressed as means ± standard deviation (x ± s), and One-way ANOVA analysis was performed to compare multiple groups. While continuous variables with skewed distribution were shown as medians (interquartile ranges), the Kruskal–Wallis rank-sum test was conducted to compare multiple groups. All categorical variables were expressed as relative numbers, the groups were compared using c2 test. Pearson correlation analysis was performed to investigate the correlation between ω-3, PTG and various risk factors of NAFLD. The effects of ω-3 and PTG on NAFLD were analyzed by multivariate logistic regression. The indirect effect was tested using the non-parametric percentile Bootstrap method with bias correction which was conducted to explore whether PTG mediated the association between ω-3 and NAFLD. The number of repeated sampling was set at 5, 000 times. P values < 0.05 were considered statistically significant.

## 3 Results

**FIGURE 1:**
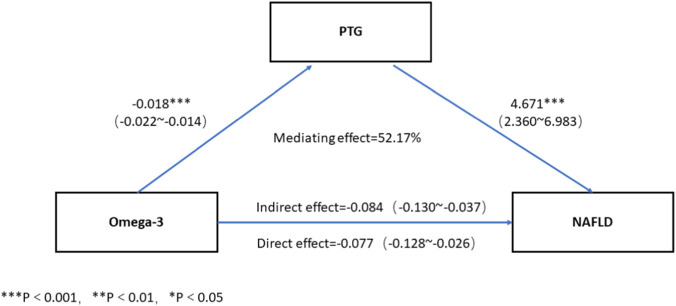
Mediating effect analysis of PTG on the association between Omega-3 and NAFLD.

A total of 239 participants were registered in this study from March to June 2024. After excluding 131 participants because of exclusion criteria, the final study group consisted of 108 participants, including 49 (45.37%) males and 59 (54.63%) females (Figure 1).

### 3.1 Clinical characteristics of the participants

A total of 108 participants ranged from 18 to 60 years old in the final analysis. The average age was 44.83 ± 9.63 years old. The incidence of NAFLD was 51.9% (56/108). The WC, Weight, BMI, TG, FINS, HOMA-IR, PTG and NAFLD prevalence were significantly different in the O1∼O4 groups (P < 0.05). The difference in Gender, Age, HC, Height, SBP, DBP, TC, LDL-C, HDL-C, Non-HDL, FPG between the four groups was not statistically significant (P > 0.05) (Table 1).

**TABLE 1:** Comparison of basic demographic characteristics in quartile groups of ω-3.

	O1 (n = 27)	O2 (n = 27)	O3 (n = 27)	O4 (n = 27)	P
Gender					0.727
Male〔n (%)〕	10 (37)	14 (52)	12 (44)	13 (48)	
Female〔n (%)〕	17 (63)	13 (48)	15 (56)	14 (52)	
Age (years)	45.59 ± 1.60	45.78 ± 1.93	47.63 ± 1.55	43.89 ± 1.67	0.569
WC (cm)	93.30 ± 1.58	87.78 ± 1.84	84.44 ± 2.16	80.00 ± 1.94	<0.001
HC (cm)	102.33 ± 1.46	100.93 ± 1.36	98.56 ± 1.64	97.30 ± 1.65	0.053
Height (cm)	164.07 ± 1.50	167.59 ± 1.23	166.04 ± 1.70	165.41 ± 1.24	0.458
Weight (cm)	75.83 ± 1.82	72.44 ± 2.01	68.59 ± 2.67	66.39 ± 2.07	0.020
BMI (kg/m2)	28.16 ± 0.53	25.81 ± 0.71	24.73 ± 0.74	24.18 ± 0.61	<0.001
SBP (mmHg)	125.89 ± 2.25	123.15 ± 1.88	120.81 ± 2.24	121.07 ± 2.05	0.214
DBP (mmHg)	80.41 ± 1.67	77.63 ± 1.21	77.48 ± 1.48	77.33 ± 1.49	0.527
TC (mmol/L)	4.34 ± 0.08	4.45 ± 0.09	4.53 ± 0.09	4.32 ± 0.09	0.305
TG (mmol/L)	1.26 ± 0.05	1.10 ± 0.06	0.89 ± 0.06	0.85 ± 0.06	<0.001
LDL-C (mmol/L)	3.14 ± 0.09	3.05 ± 0.09	3.08 ± 0.09	2.90 ± 0.09	0.222
HDL-C (mmol/L)	1.04 ± 0.05	1.25 ± 0.08	1.17 ± 0.07	1.15 ± 0.06	0.146
Non-HDL (mmol/L)	3.29 ± 0.09	3.21 ± 0.10	3.35 ± 0.08	3.17 ± 0.09	0.745
FPG (mmol/L)	5.26 ± 0.10	5.11 ± 0.10	5.10 ± 0.09	5.18 ± 0.09	0.455
FINS (μIU/mL)	16.81 ± 0.99	14.06 ± 1.25	10.31 ± 1.26	9.27 ± 1.03	<0.001
HOMA-IR	3.96 ± 0.27	3.26 ± 0.34	2.40 ± 0.32	2.18 ± 0.26	<0.001
PTG (mmol/L)	2.49 ± 0.13	2.06 ± 0.13	1.34 ± 0.07	1.23 ± 0.08	<0.001
NAFLD [n (%)]	27 (100)	20 (74)	8 (30)	1 (4)	<0.001

WC, waist circumference; HC, hip circumference; BMI, body mass index; SBP, systolic blood pressure; DBP, diastolic blood pressure; TC, total cholesterol; TG, triglyceride; LDL-C, low density lipoprotein cholesterol; HDL-C, high density lipoprotein cholesterol; Non-HDL, non-HDL, cholesterol; FINS, fasting insulin; FPG, fasting plasma glucose; HOMA-IR, homeostasis model assessment of insulin resistance; PTG, postprandial triglyceride; NAFLD, non-alcoholic fatty liver disease. Data are means ± standard deviations or medians (interquartile ranges) for continuous variables, and numbers (proportions) for categorical variables. P values are calculated by One-way ANOVA, analysis and Kruskal–Wallis rank-sum test for continuous variables, Chi-square test for categorical variables. P < 0.05 was considered statistically significant.

The levels of WC, HC, Weight, BMI, SBP, TG, LDL-C, FINS, HOMA-IR, ω-3 and NAFLD prevalence were significantly different in the P1∼P4 groups (P < 0.05). The difference in Gender, Age, Height, DBP, TC, HDL-C, Non-HDL, FPG between the four groups was not statistically significant (P > 0.05).Moreover, the prevalence of NAFLD increased with elevated PTG and decreased with elevated ω-3 (P < 0.05) (Table 2).

**TABLE 2:** Comparison of basic demographic characteristics in quartile groups of PTG.

	P1 (n = 27)	P2 (n = 28)	P3 (n = 26)	P4 (n = 27)	P
Gender					0.338
Male〔n (%)〕	10 (37)	13 (46)	10 (38)	16 (59)	
Female〔n (%)〕	17 (63)	15 (54)	16 (62)	11 (41)	
Age (years)	43.33 ± 1.3	47.00 ± 1.91	47.35 ± 1.54	45.22 ± 1.84	0.317
WC (cm)	77.67 ± 1.99	86.57 ± 1.88	89.31 ± 1.75	92.0 ± 1.69	<0.001
HC (cm)	94.48 ± 1.85	100.71 ± 1.35	102.00 ± 1.32	101.96 ± 1.24	<0.001
Height (cm)	164.07 ± 1.59	166.25 ± 1.31	165.73 ± 1.56	167.04 ± 1.27	0.513
Weight (cm)	62.31 ± 2.49	69.90 ± 1.88	74.99 ± 1.93	76.23 ± 1.65	<0.001
BMI (kg/m2)	22.97 ± 0.67	25.27 ± 0.59	27.31 ± 0.61	27.39 ± 0.63	<0.001
SBP (mmHg)	119.22 ± 2.21	121.14 ± 1.97	123.15 ± 2.35	127.48 ± 1.66	0.034
DBP (mmHg)	75.74 ± 1.47	77.50 ± 1.38	79.35 ± 1.23	80.33 ± 1.69	0.198
TC (mmol/L)	4.47 ± 0.10	4.47 ± 0.07	4.47 ± 0.10	4.24 ± 0.07	0.161
TG (mmol/L)	0.77 ± 0.05	0.91 ± 0.05	1.12 ± 0.06	1.31 ± 0.05	<0.001
LDL-C (mmol/L)	2.84 ± 0.07	3.13 ± 0.10	3.14 ± 0.10	3.06 ± 0.09	0.024
HDL-C (mmol/L)	1.26 ± 0.06	1.19 ± 0.06	1.10 ± 0.07	1.06 ± 0.06	0.169
Non-HDL (mmol/L)	3.21 ± 0.08	3.28 ± 0.10	3.36 ± 0.10	3.18 ± 0.08	0.521
FPG (mmol/L)	4.99 ± 0.09	5.23 ± 0.09	5.26 ± 0.10	5.17 ± 0.11	0.137
FINS (μIU/mL)	7.69 ± 0.79	11.74 ± 1.19	14.69 ± 1.47	16.45 ± 0.88	<0.001
HOMA-IR	1.76 ± 0.20	2.77 ± 0.30	3.49 ± 0.39	3.80 ± 0.24	<0.001
ω-3 (μmol/L)	125.36 ± 4.81	114.68 ± 4.00	94.46 ± 4.16	82.36 ± 1.98	<0.001
NAFLD [n (%)]	0	8 (29)	21 (81)	27 (100)	<0.001

WC, waist circumference; HC, hip circumference; BMI, body mass index; SBP, systolic blood pressure; DBP, diastolic blood pressure; TC, total cholesterol; TG, triglyceride; LDL-C, low density lipoprotein cholesterol; HDL-C, high density lipoprotein cholesterol; Non-HDL, non-HDL, cholesterol; FINS, fasting insulin; FPG, fasting plasma glucose; HOMA-IR, homeostasis model assessment of insulin resistance; PTG, postprandial triglyceride; NAFLD, non-alcoholic fatty liver disease; ω-3, Omega-3, levels. Data are means ± standard deviations or medians (interquartile ranges) for continuous variables, and numbers (proportions) for categorical variables. P values are calculated by One-way ANOVA, analysis and Kruskal–Wallis rank-sum test for continuous variables, Chi-square test for categorical variables. P < 0.05 was considered statistically significant.

### 3.2 Pearson correlation analysis of ω-3, PTG and basic indicators

Pearson correlation analysis indicated that ω-3 was negatively correlated with WC (r = −0.437, P < 0.001), HC (r = −0.276, P = 0.004), Weight (r = −0.370, P < 0.001), BMI (r = −0.431, P < 0.001), TG (r = −0.387, P < 0.001), FINS (r = −0.399, P < 0.001), HOMA-IR (r = −0.358, P < 0.001), the prevalence of NAFLD (r = −0.686, P < 0.001). Similarly, PTG was positively correlated with WC (r = 0.402, P < 0.001), HC (r = 0.275, P = 0.004), Weight (r = 0.365, P < 0.001), BMI (r = 0.376, P < 0.001), TG (r = 0.667, P < 0.001), FINS (r = 0.450, P < 0.001), HOMA-IR (r = 0.413, P < 0.001), the prevalence of NAFLD (r = 0.729, P < 0.001), but negatively correlated with HDL-C (r = −0.195, P < 0.001) and ω-3 (r = −0.615, P < 0.001) (Table 3).

**TABLE 3:** Pearson correlation analysis of ω-3, PTG and basic physical and biochemical indicators.

	ω-3		PTG	
	r	P	r	P
Gender	−0.008	0.935	0.070	0.470
Age (years)	−0.064	0.507	0.059	0.546
WC (cm)	−0.437**	<0.001	0.402**	<0.001
HC (cm)	−0.276**	0.004	0.275**	0.004
Height (cm)	−0.021	0.827	0.116	0.233
Weight (cm)	−0.370**	<0.001	0.365**	<0.001
BMI (kg/m2)	−0.431**	<0.001	0.376**	<0.001
SBP (mmHg)	−0.084	0.389	0.226*	0.019
DBP (mmHg)	−0.080	0.413	0.186	0.053
TC (mmol/L)	0.035	0.716	−0.113	0.244
TG (mmol/L)	−0.387**	<0.001	0.667**	<0.001
LDL-C (mmol/L)	−0.163	0.091	0.174	0.073
HDL-C (mmol/L)	0.089	0.360	−0.195*	0.044
Non-HDL (mmol/L)	−0.029	0.765	0.030	0.759
FPG (mmol/L)	−0.044	0.651	0.116	0.232
FINS (μIU/mL)	−0.399**	<0.001	0.450**	<0.001
HOMA-IR	−0.358**	<0.001	0.413**	<0.001
ω-3 (μmol/L)	-	-	−0.615**	<0.001
NAFLD [n (%)]	−0.686**	<0.001	0.729**	<0.001

WC, waist circumference; HC, hip circumference; BMI, body mass index; SBP, systolic blood pressure; DBP, diastolic blood pressure; TC, total cholesterol; TG, triglyceride; LDL-C, low density lipoprotein cholesterol; HDL-C, high density lipoprotein cholesterol; Non-HDL, non-HDL, cholesterol; FINS, fasting insulin; FPG, fasting plasma glucose; HOMA-IR, homeostasis model assessment of insulin resistance; ω-3, Omega-3, levels; NAFLD, non-alcoholic fatty liver disease. P < 0.05 was considered statistically significant.

### 3.3 Multivariate logistic regression analysis of the association between ω-3 and the risk of NAFLD

ω-3 as an independent variable was assigned with NAFLD as a dependent variable (assignment: yes = 1, no = 0). Logistic analysis indicated that ω-3 was negatively associated with NAFLD prevalence without adjusting confounding factors (P < 0.001) (Model 1). Compared to the low ω-3 group, the risk of NAFLD significantly decreased in high ω-3 group〔OR = 0.030, 95%CI (0.010, 0.087)〕, after adjustment for age and gender, the risk of NAFLD still significantly decreased in high ω-3 group〔OR = 0.025, 95%CI (0.008, 0.078)〕 (P < 0.001) (Model 2). After further adjustment for WC, HC, BMI, SBP, DBP, height, weight, the risk of NAFLD still significantly decreased in high ω-3 group, that is, the risk of developing NAFLD in the high-ω-3 group was only 2.4% compared to the low-ω-3 group.〔OR = 0.024, 95%CI (0.006, 0.104)〕(Model 3) (Table 4).

**TABLE 4:** Multivariate logistic regression analysis of the association between ω-3 and the risk of NAFLD.

	Model 1		Model 2		Model 3	
	OR (95%CI)	P	OR (95%CI)	P	OR (95%CI)	P
low ω-3 group	1	-	1	-	1	-
high ω-3 group	0.030 (0.010–0.087)	<0.001	0.025 (0.008–0.078)	<0.001	0.024 (0.006–0.104)	<0.001

Model 1 was not adjusted for confounding factors. Model 2 was adjusted for age and gender. Model 3 was adjusted for the covariates of model 2 plus waist circumference, hip circumference, body mass index, systolic blood pressure, diastolic blood pressure, height, weight. Odds ratios and 95% CIs, were calculated per 1-SD, increment of Omega-3; OR, odds ratio; CI, confidence interval.

### 3.4 Multivariate logistic regression analysis of the association between PTG and the risk of NAFLD

PTG as independent variables were assigned with the risk of NAFLD as a dependent variable (assignment: yes = 1, no = 0), and multivariate logistic analysis was conducted. The results showed that a positive relationship could be found between PTG and the risk of NAFLD without adjusting confounding factors. For every 0.1 mmol/L increase in PTG level, the risk of NAFLD increases by approximately 71%.〔OR = 1.710, 95%CI (1.398–2.090)〕and after adjustment for age, gender, WC, HC, Height, Weight, SBP, DBP, BMI〔OR = 1.760, (1.373–2.256)〕(P < 0.001) (Model 1 and Model 2). After further adjustment for TC, TG, LDL-C, HDL-C, Non-HDL, FPG, FINS, HOMA-IR, 〔OR = 5.351 (1.270–22.537)〕(Model 3). In this logistic regression model, the odds ratio represents the corresponding risk change when this variable increases by 0.1 mmol/L (Table 5).

**TABLE 5:** Multivariate logistic regression analysis of the association between Omega-3, PTG and the risk of NAFLD.

	Model 1		Model 2		Model 3	
	OR (95%CI)	P	OR (95%CI)	P	OR (95%CI)	P
PTG	1.710 (1.398–2.090)	<0.001	1.760 (1.373–2.256)	<0.001	5.351 (1.270–22.537)	0.020

Model 1 was not adjusted for confounding factors. Model 2 was adjusted for age, gender, waist circumference, hip circumference, body mass index, systolic blood pressure, diastolic blood pressure, height, weight.Model 3 was adjusted for the covariates of model 2 plus total cholesterol, triglyceride, low density lipoprotein cholesterol, high density lipoprotein cholesterol, non-HDL, cholesterol, fasting insulin, fasting plasma glucose, Homeostasis Model Assessment of Insulin Resistance. Odds ratios and 95% CIs, were calculated per 1-SD, increment of PTG; OR, odds ratio; CI, confidence interval.

### 3.5 Mediating effect analysis of PTG on the association between Omega-3 and the risk of NAFLD

This study showed that both ω-3 and PTG were associated with NAFLD. Moreover ω-3 was negatively associated with PTG. It suggested that PTG could potentially mediate the association between ω-3 and NAFLD. To explore whether PTG mediated the association between ω-3 and NAFLD, we performed a mediation effect analysis using the bootstrap method. The results showed that ω-3 had a significant direct effect on NAFLD risk 〔β = −0.077, 95%CI (−0.128, −0.026)〕, and PTG partially mediated the indirect effect of ω-3 on NAFLD 〔β = −0.084, 95%CI (−0.130, −0.037)〕. The mediating effect accounted for 52.17% of the total effect (Figure 1).

## 4 Discussion

Omega-3 is an important type of polyunsaturated fatty acid that the human body requires, and it is closely related to lipid metabolism. The present study’s novelty is applying the omega-3 levels as a predictor of NAFLD risk, which could provide more information on the association between dietary intake and NAFLD. This study indicated that in individuals with normal fasting lipid levels, the levels of WC, Weight, BMI, TG, FINS, HOMA-IR, PTG and NAFLD prevalence decreased with elevated omega-3 levels. Multivariate logistic regression analysis showed that omega-3 levels was negatively correlated with NAFLD when no confounding factors were adjusted. After adjusting for multiple confounding factors, a significant negative correlation was observed between NAFLD risk and the omega-3 levels. It suggested that a lower omega-3 level was a risk factor for NAFLD prevalence. This complemented clinical evidence for omega-3 assessing NAFLD risk. Research has found that insufficient levels of EPA and DHA in the liver may cause the balance to lean towards liver fatty acid production; indeed, the relative levels of EPA and DHA in the livers of patients with NAFLD have been found to be lower than those in a control group (Musa-Veloso et al., 2018). The balance between ω-3 and ω-6 PUFA is crucial for regulating the amount of lipids in the liver (Shama and Liu, 2020), which was basically consistent with the results of this study. This study validates the fact that ω-3 PUFA plays an important role in regulating fat accumulation and fat elimination in the liver.

The underlying mechanism of the association between the ω-3 and NAFLD was not fully understood. The possible mechanism was as follows: elevated serum ω-3 can block the fatty acid synthesis pathway by down-regulating the expression of the SREBP-1c gene. SREBP-1 is the key transcription factors for fat production (Dias et al., 2022). Furthermore, elevated ω-3 may also lead to inhibit the activity of FASN, which is responsible for the synthesis and storage of triglycerides (Ferreira et al., 2024). At the same time, ω-3 act as natural ligands for PPARα, directly binding to and activating this receptor. This significantly enhances the activity of the downstream gene carnitine CPT1, which transports long-chain fatty acids into mitochondria and is a key enzyme in fatty acid β-oxidation (Ulucay Kestane and Bas, 2024). Studies have shown that increasing the intake of ω-3 is highly effective in reducing PTG levels in healthy individuals (Austin et al., 2021). High PTG is positively related to a higher risk of NAFLD (Hou et al., 2021), Therefore, this study hypothesized that the ω-3 improves NAFLD by reducing PTG. This study has confirmed this view.Therefore, it has become a therapeutic drug with great potential for treating NAFLD. It also verifies that dietary intake of omega-3 play an essential role in the pathogenesis of NAFLD.

Most scholars believe that NAFLD is more closely related to fasting blood lipids (Ulucay Kestane and Bas, 2024); therefore, fasting blood lipids are often used for the assessment. However, in clinical practice, many patients with NAFLD have normal fasting blood lipid levels. Therefore, we believe that simply using fasting blood lipid levels is insufficient to comprehensively assess lipid metabolism and predict the risk of NAFLD. Postprandial blood lipid levels have been receiving increasing attention because they compensate for the shortcomings of simple fasting blood lipid tests. Some studies suggested that high PTG is positively related to a higher risk of NAFLD, and the PTG concentrations of patients with NAFLD are higher than in healthy individuals (Hou et al., 2021). In this study, PTG was positively correlated with the prevalence of NAFLD, which confirmed the view above. Logistic regression analysis demonstrated that when per 1 increment of PTG, the risk of NAFLD also significantly increased.

Further mediation effect analysis indicates that PTG statistically played a mediating role to some extent, thereby explaining the association between ω-3 and NAFLD, which indicated that PTG may be the reason for the correlation between ω-3 and NAFLD. Tinker proposed that ω-3 may reduce PTG by inhibiting the synthesis and secretion of ApoB in the liver and intestines (Tinker et al., 1999). In addition, it enhanced the clearance of CM-TG by increasing LPL activity (Park and Harris, 2003). It provided a clinical basis for the prevention and treatment of NAFLD. In addition, insulin resistance may also mediate the association between ω-3 and NAFLD.

This study also has several limitations. Firstly, The cross-sectional design hinders the inference of causal relationships. Secondly, The sample size of this study is relatively small, which may increase the risks of selection bias and confounding bias. The subjects in this study were selected from those with normal fasting blood lipid levels, Future studies will need to validate these findings in community populations or patient cohorts with greater representativeness and a broader metabolic spectrum to assess their generalizability. Additionally, Although we adjusted for multiple covariates, unmeasured factors (such as physical activity, diet, alcohol intake, etc.) may still affect the results. The lack of detailed lifestyle assessment may lead to residual confounding factors. Future research will require conducting prospective cohort studies or randomized controlled trials with larger sample sizes to verify the causal relationships of these associations.

## 5 Conclusion

ω-3 is an independent protective factor for NAFLD, and PTG statistically partially mediates the effect of ω-3 on NAFLD. Regular monitoring of ω-3 and PTG has a significant clinical significance for evaluating NAFLD and related diseases. Simultaneously, it providing new evidence for the application of ω-3 in improving NAFLD in clinical work.

## Data Availability

The original contributions presented in the study are included in the article/Supplementary Material, further inquiries can be directed to the corresponding author.
